# Purification and Properties of a Plasmin-like Marine Protease from Clamworm (*Perinereis aibuhitensis*)

**DOI:** 10.3390/md22020068

**Published:** 2024-01-27

**Authors:** Tingting Jiang, Bing Zhang, Haixing Zhang, Mingjun Wei, Yue Su, Tuo Song, Shijia Ye, Yuping Zhu, Wenhui Wu

**Affiliations:** 1 Department of Marine Biopharmacology, College of Food Science and Technology, Shanghai Ocean University, Shanghai 201306, China; j15037793607@163.com (T.J.); bling9803@163.com (B.Z.); starfishhx@163.com (H.Z.); weimj98@163.com (M.W.); suyue0616@163.com (Y.S.); 15026952710@163.com (T.S.); yyyesj@163.com (S.Y.); 2 Marine Biomedical Science and Technology Innovation Platform of Lin-gang Special Area, Lane 218, Haiji Sixth Road, Shanghai 201306, China; 3 Basic Medical Experimental Teaching Center, Basic Medical College, Naval Medical University, Shanghai 200433, China; 4 East China Sea Marine Biological Resources Engineering Technology Center, Zhongke Road, Putuo District, Zhoushan 316104, China

**Keywords:** *Perinereis aibuhitensis*, plasmin-like protease, amino acid residue sequences, fibrinolysis in vitro

## Abstract

Marine organisms are a rich source of enzymes that exhibit excellent biological activity and a wide range of applications. However, there has been limited research on the proteases found in marine mudflat organisms. Based on this background, the marine fibrinolytic enzyme FELP, which was isolated and purified from clamworm (*Perinereis aibuhitensis*), has exhibited excellent fibrinolytic activity. We demonstrated the FELP with a purification of 10.61-fold by precipitation with ammonium sulfate, ion-exchange chromatography, and gel-filtration chromatography. SDS-PAGE, fibrin plate method, and LC–MS/MS indicated that the molecular weight of FELP is 28.9 kDa and identified FELP as a fibrinolytic enzyme-like protease. FELP displayed the maximum fibrinolytic activity at pH 9 (407 ± 16 mm^2^) and 50 °C (724 ± 27 mm^2^) and had excellent stability at pH 7–11 (50%) or 30–60 °C (60%), respectively. The three-dimensional structure of some amino acid residues of FELP was predicted with the SWISS-MODEL. The fibrinolytic and fibrinogenolytic assays showed that the enzyme possessed direct fibrinolytic activity and indirect fibrinolysis via the activation of plasminogen; it could preferentially degrade Aα-chains of fibrinogen, followed by Bβ- and γ-chains. Overall, the fibrinolytic enzyme was successfully purified from *Perinereis aibuhitensis,* a marine Annelida (phylum), with favorable stability that has strong fibrinolysis activity in vitro. Therefore, FELP appears to be a potent fibrinolytic enzyme with an application that deserves further investigation.

## 1. Introduction

The ocean is a large potential source of bioactives [1,2]. Due to the particular living environment, marine organisms have generated bioactive substances with significant specialization [3], especially enzymes, which have properties related to their living environment, such as thermal stability, salt tolerance, cold adaptation, etc., and offer good prospects for industrial applications [4] and biomedicine [5]. Marine organisms are important resources for the production of enzymes, and through isolation and purification from marine microorganisms, a variety of enzymes have been obtained, which exhibit antimicrobial activity [3,6,7,8], hydrolytic activity against blood proteins [9], collagen solubilizing activity [10] and hydrolysis of alginate for efficient production of alginate oligosaccharides [11], and others. In addition to microorganisms, there are also a wide variety of enzymes, such as cellulases [12] and digestive enzymes [13], in marine animals and plants.

It is noteworthy that enzymes with fibrinolytic activity are found in marine animals as well as in microorganisms. Marine microorganisms of the genus *Bacillus* and *Aspergillus* are considered an important source of fibrinolytic enzyme production [4,14,15,16]. The fibrinolytic enzymes derived from the fungus demonstrate promising thrombolytic and anticoagulant activities [17]. The purified protease production by the strain *Aspergillus brasiliensis* BCW2 has de-clotting and blood stain removal properties [18]. Che et al. (2020) successfully employed, for the high production of fibrinolytic enzymes, fibase from marine *Bacillus subtilis*, which was expressed in *Komagataella phaffii* GS115 [19]. Members of Annelida (phylum) and Echinodermata in marine organisms contain proteases with fibrinolytic and anticoagulant effects; e.g., Qingqing Bi et al. (2013) purified a fibrinolytically active UFEII from marine echiuroid worms that exhibits fibrin-direct solubilizing and fibrinogen-activating activities [20]. Yahui Ge et al. (2018) purified a novel antithrombotic enzyme from *Sipunculus nudus*, which could improve the coagulation system and inhibit thrombus formation [21]. Marine proteases have shown a good potential for application due to their high safety profile, a wide range of biological activities, and natural origin in the past decades. However, little attention has been paid to the marine mudflat organisms known as *Perinereis aibuhitensis (P. aibuhitensis)*, which also belong to Annelida (phylum). They are often present as bait in aquaculture [22]. They have been explored as research subjects to investigate the potential impacts of marine pollutants [23]. The *P. aibuhitensis* extract obtained in one study exhibited antithrombotic activity, but the specific substances responsible for fibrinolytic effects were not identified [24]. Therefore, it is necessary to investigate the fibrinolytically active components in the *P. aibuhitensis* extract.

In this study, FELP (a protease with plasmin-like activity) was isolated and purified from Annelida (phylum), *P. aibuhitensis*, in a marine mudflat. The amino acid residues were analyzed, and it was confirmed to possess fibrinolytic activity in vitro. FELP shows promise in treating and preventing thrombosis due to its exceptional stability and fibrinolytic activity. Collectively, these results provide the direction and theoretical basis for the development of marine fibrinolytic enzymes.

## 2. Results

### 2.1. Isolation and Purification of FELP with Activity Monitoring

**Figure 1:**
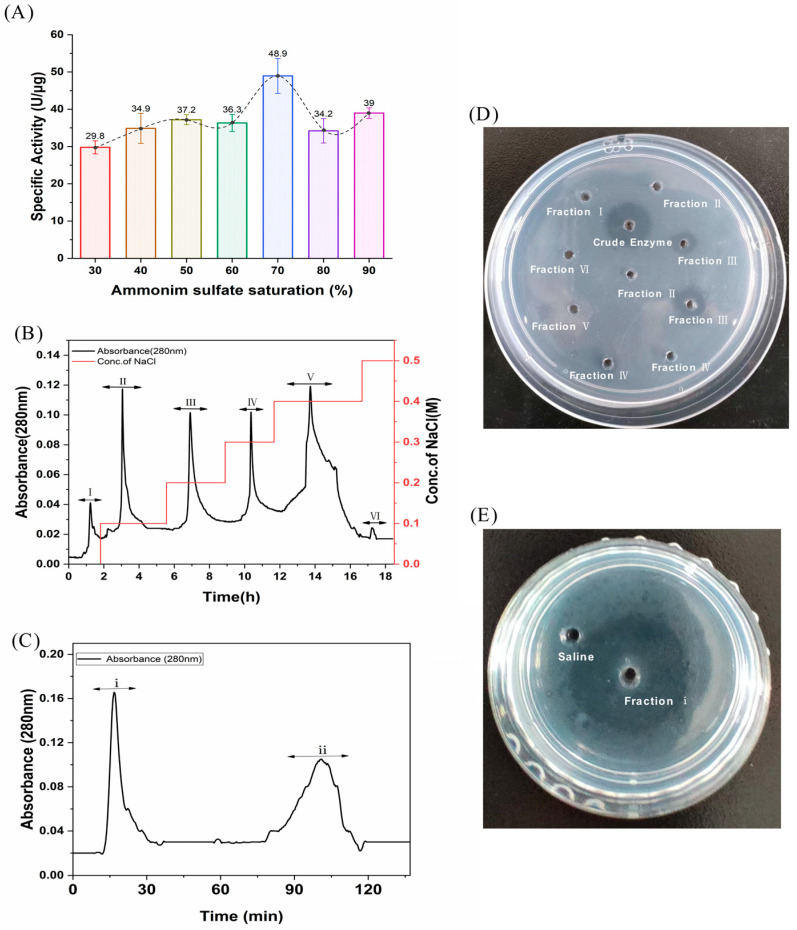
Isolation and purification of FELP from *P. aibuhitensis*. (**A**) The isolation of fibrinolytic enzyme from supernatant through ammonium sulfate precipitation; (**B**) An ion-exchange chromatography of crude enzyme (200 mg) with DEAE-sepharose FF column; (**C**) Gel-filtration chromatography of fraction III (1 g) with Superdex G-50. (**D**) The fibrinolytic activity of fraction I–VI. (**E**) The fibrinolytic activity of fraction i.

FELP from *P. aibuhitensis* was isolated and purified using the purification process mentioned later. The activity of the fractions obtained from each isolation and purification step was monitored using fibrinolysis as an evaluation index. The relationship between the activity of the samples obtained at each ammonium sulphate saturation gradient and ammonium sulfate saturation is shown in Figure 1A. Obviously, the highest enzyme activity was found at 70% ammonium sulphate saturation, so 70% was chosen as the optimum ammonium sulphate saturation for the extraction of FELP.

The crude enzyme was re-dissolved in Tris-HCl (0.02 M), filtered through a 0.45 µm membrane, and then applied to the DEAE-sepharose FF column. The crude sample was eluted with 0–0.5 M NaCl, and the mixtures of protease samples were collected at 280 nm to obtain six fractions (I, II, III, IV, V, and VI) with the DEAE-sepharose FF column (Figure 1B). Surprisingly, fraction III was found to have better fibrinolytic activity according to the fibrin plate than the other fractions (Figure 1D). Fraction III was then dialyzed, freeze-dried, and carried with the Sephadex G-50 column. The first peak (fraction i) in Figure 1B showed excellent fibrinolytic activity. Throughout the purification process, FELP from *P. aibuhitensis* was purified 10.61-fold. The active fractions of the purification process were assayed for fibrinolytic activity and corresponding protein amount (Table 1).

### 2.2. SDS-PAGE

**Figure 2:**
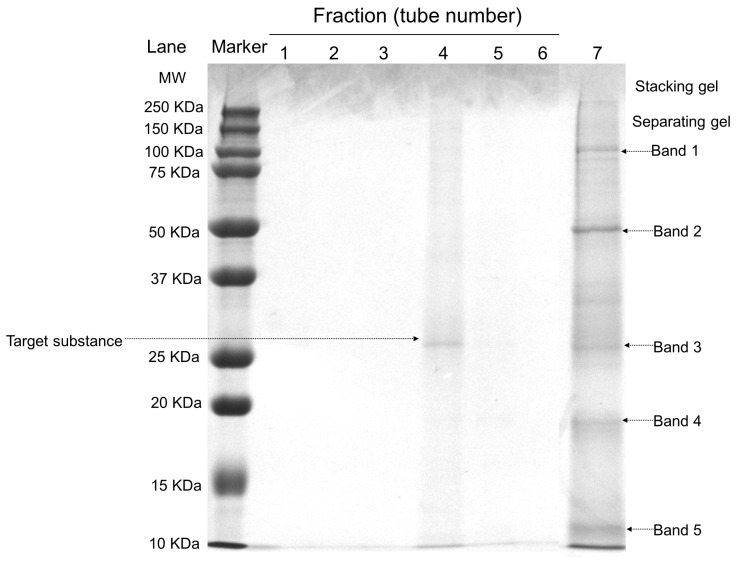
SDS-PAGE analysis of the purified enzyme. The crude extract and the fractions were separated by 12.5% SDS-PAGE; the bands were stained with Coomassie brilliant blue R-250 and then were transferred to the decolorizing fluid (V_Ethanol_:V_Acetic acid_:V_H2O_ = 2:1:7). Lanes: Marker, protein marker (10–250 kDa); 1–6, Different tubes of elution collected in fraction i (50 mg/mL); 7, The crude exact of *P. aibuhitensis* (5 mg/mL).

The five main bands in the crude extract were purified into a single band (Figure 2). The molecular weight analysis of FELP showed 28.9 kDa by comparing the relative mobility of the enzyme to a marker. According to the previous reports, the molecular weight of proteases with fibrinolytic activity ranges from 14 to 97 kDa with an average of 27–29 kDa [25]. The molecular weight of FELP was within this interval. Furthermore, the first peak of Superdex G-50 was protein-free in all tubes except tube 4.

### 2.3. Biochemical Properties of the Purified Fibrinolytic Enzyme

#### 2.3.1. Effect of Inhibitors

**Figure 3:**
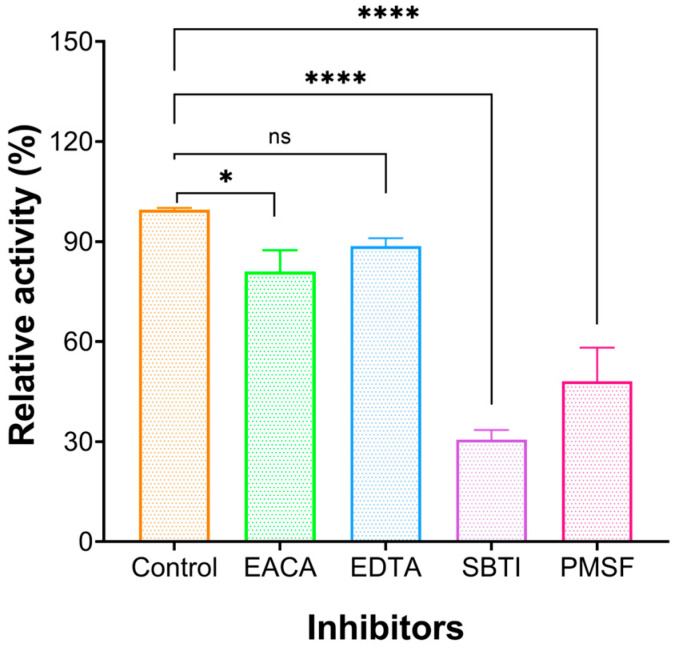
Effect of protease inhibitors on the activity of FELP. FELP (50 mg/mL) was mixed with EACA (10 mM), EDTA (5 mM), SBTI (5 mM), and PMSF (5 mM) at 37 °C, pH 7.4 for 18 h. The data represent mean ± SD, *n* = 3. *, and ns: not significant, * *p* < 0.05, and **** *p* < 0.0001 compared with control, respectively.

The enzyme activity of FELP was affected by different protease inhibitors: soybean trypsin inhibitor (SBTI), phenylmethanesulfonyl fluoride (PMSF) at 5 mM concentration (Figure 3), ethylene diamine tetraacetic acid (EDTA) slightly inhibited the enzyme activity at 5 mM, and epsilon amino caproic acid (EACA) still weakly inhibited protease activity at 10 mM. The enzyme activity was significantly inhibited by SBTI and serine protease inhibitor-like PMSF.

#### 2.3.2. Effect of Temperature and Thermal Stability of FELP

**Figure 4:**
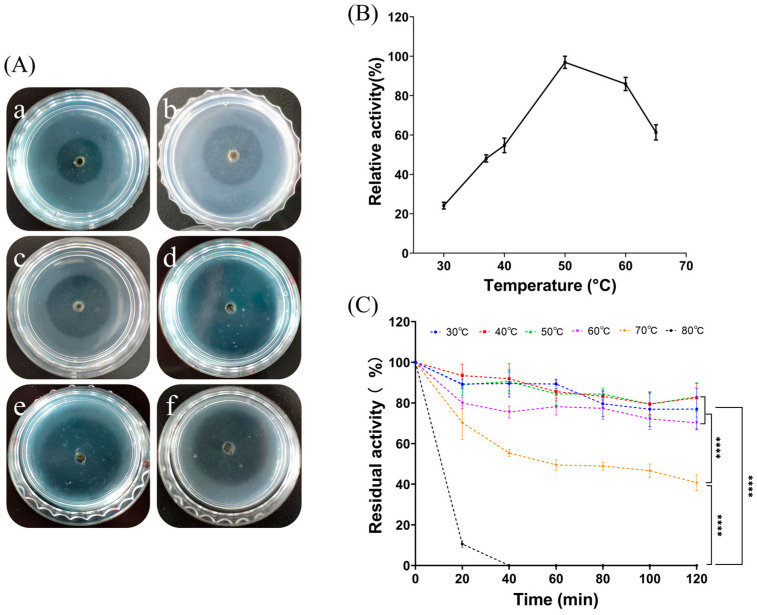
Optimum temperature and thermal stability were determined by evaluating the enzyme activity at different temperatures. Formation of transparent circles (**A**) and relative enzyme activity (**B**) of FELP (50 mg/mL) at 30–65 °C ((**a**–**f**) in (**A**) correspond to 30 °C, 37 °C, 40 °C, 50 °C, 60 °C, and 65 °C, respectively). (**C**) Determination of changes in fibrinolytic activity with incubation at different temperatures for 0, 20, 40, 60, 80, 100, and 120 min. The data represent mean ± SD, *n* = 3. **** indicate *p* < 0.0001 compared with each other in any groups.

From Figure 4A, it is clear that the transparent circle of fibrinolysis in the fibrin plates displayed a trend of increasing and then decreasing as the temperature increased. As shown in Figure 4B, the enzyme activity was highest when the temperature reached 50 °C; as the holding time increased, the protease activity decreased. The higher the temperature, the worse was the protease stability. FELP has better temperature stability at 30 to 60 °C; even if incubated for 2 h, it still maintained more than 60% of the protease viability (Figure 4C). Furthermore, following incubation for 2 h at each temperature, the residual activity of FELP was significantly reduced at 70 °C and 80 °C compared to the other temperatures.

#### 2.3.3. Effect of pH and Stability of FELP

**Figure 5:**
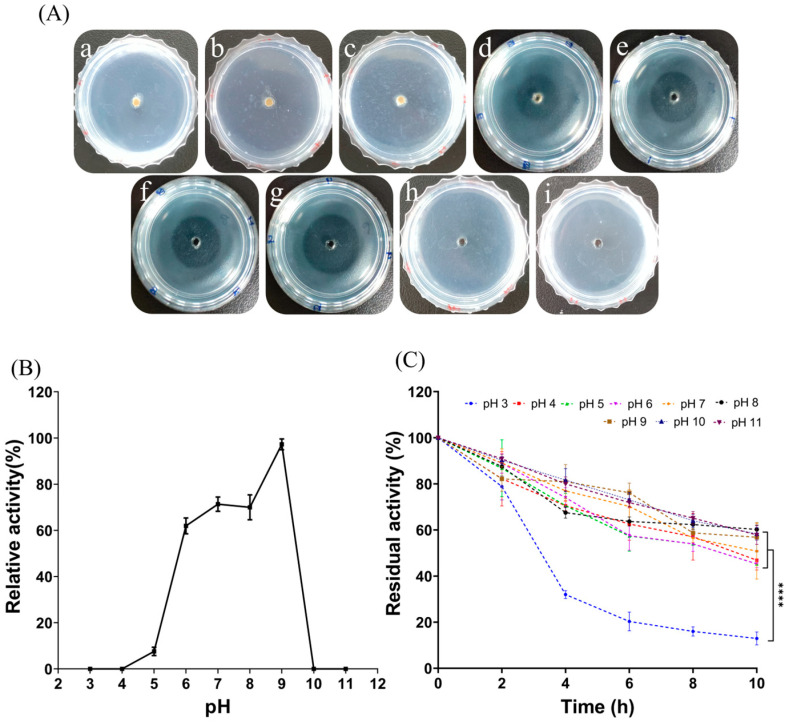
Influence of pH on the fibrinolytic activity of FELP (50 mg/mL) with different buffers (**B**) and the transparent circles in the fibrin plate (**A**) ((**a**–**i**) in (**A**) correspond to pH 3–11, respectively). (**C**) Determination of changes in fibrinolytic activity with incubation in different buffers for 0, 2, 4, 6, 8, and 10 h. The data represent mean ± SD, *n* = 3. **** indicate *p* < 0.0001 compared with each other in any groups.

The influence of pH on enzyme activity was measured by treating with a pH of 3, 4, 5, 6, 7, 8, 9, 10, and 11 using different buffers (Figure 5A). The optimal enzyme activity for FELP was pH 9; either peracid or peralkaline environments inactivated the fibrinolytic enzymes (Figure 5B). At pH 7–11, the activity of FELP slowly decreased and remained above 50% for 10 h. At pH 3, the activity of the protease fell below 50% after 4 h. After incubating at each pH for 10 h, the residual activity of FELP was significantly lower at pH 3 compared to the other pH levels.

### 2.4. Analysis of Amino Acid Residue Sequences of FELP

FELP was obtained from a clearly visible SDS-PAGE protein band; then, the gels with the target protein bands were reduced and alkylated for enzymatic digestion. The peptide mixture was sorted according to the polarity with a liquid chromatography column. The peptide segments were made with dots through an ESI ion source and then entered into the mass spectrometer to collect a primary mass spectrum; the ions with the highest ionic strengths from the primary mass spectrum were selected and typed into their secondary mass spectra. The secondary mass spectra obtained from the experiment were compared with the secondary mass spectra of fragment ions obtained through simulated hydrolysis and simulated fragmentation in the uniprotkb_Perinereis_245_2023_08_20 database to obtain the peptide identified results (Table 2). With the exception of VSTYMNWIGL, all peptide fragments had high scores, and we believed the identification results were genuine. Three peptides were created by joining the six peptide fragments in Table 2.

Peptide 1:

IVGGQESRPNEFPWQVSMQSSFGSHYCGAIIINRNWIMTAAHCTAGDSASDLYLMVGEHDR

Peptide 2:

TAVVSGWGTLRSGGPCCPQILQYVQVPVISNNECNTIDYPGDITDGMICAGNRLSTDACQGDSGGPLVVK

Peptide 3:

VSTYMNWIGL

**Figure 6:**
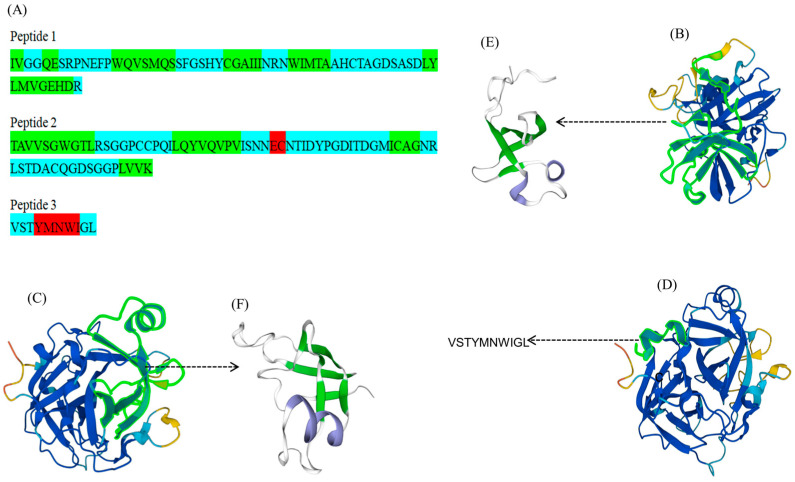
Predicted structures of peptides and B8Y626. (**A**): The predicted secondary structure of the three peptide chains (red indicates alpha helix, green indicates beta-sheet, and blue indicates coil). (**B**–**D**): The 3D structure of B8Y626 (the green fluorescently labeled fragments in (**B**–**D**) are the parts of FELP that match with mass spectrometry). (**E**,**F**): The 3D structure of peptides (green indicates beta-sheet, blue indicates alpha helix, and white indicates coil).

The identified peptides were then used to match the proteins in the database. Notably, the above peptides were matched with a protease B8Y626 from *P. aibuhitensis* (Korean lugworm), which was not reviewed. Homology modeling of the spliced peptides was performed using the SWISS-MODEL to obtain the 3D structure (Figure 6E,F). The secondary structure of the peptides had an alpha helix, beta-sheet, and coil (Figure 6A).

### 2.5. Fibrinolytic Activity

**Figure 7:**
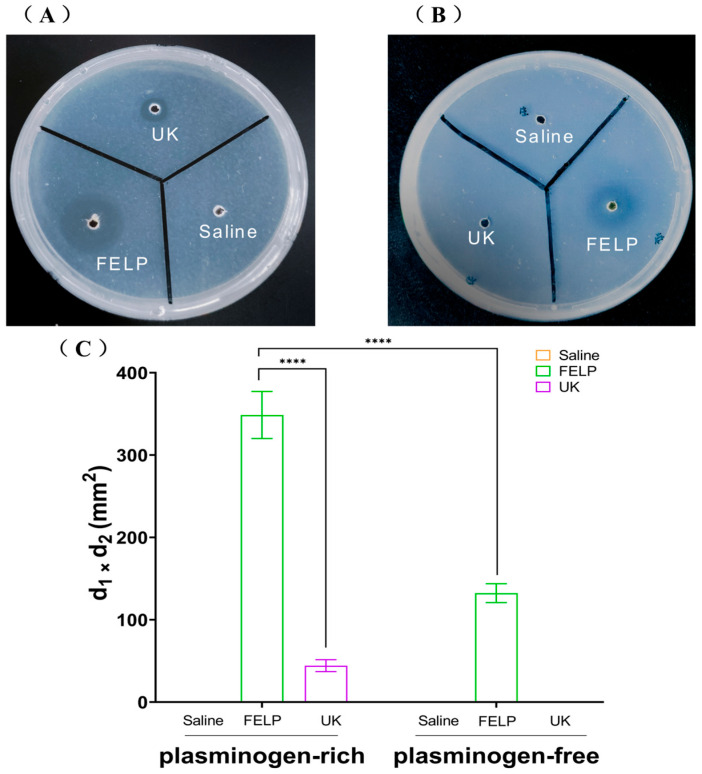
Fibrinolytic activity of FELP. The samples were applied to the wells in the plasminogen-rich fibrin plate (**A**) and plasminogen-free fibrin plate. (**B**) Saline, UK (1000 U), and FELP (50 mg/mL) were incubated at 37 °C for 18 h. (**C**) The fibrinolytic activity of saline, urokinase (1000 U), and FELP (The fibrinolytic activity was indicated by the two perpendicular diameters of the transparent circles). The data represent mean ± SD, *n* = 3. **** indicate *p* < 0.0001 between any concentration and FELP in the plasminogen-rich fibrin plate.

The fibrinolytic activity of FELP was investigated with fibrin-plate assays. Compared to saline, FELP had a distinctive transparent circle, which indicated that FELP had fibrinolytic activity. As illustrated in Figure 7A, in plasminogen-rich fibrin plates, urokinase (1000 U) and FELP both showed distinct transparent circles. On the plate that had been extinguished with fibrinogen (Figure 7B), the transparent circle of FELP was significantly smaller than that of the plate that had not been extinguished, and the edges appeared blurry. The fibrinolytic activity of FELP (348.67 ± 28.59 mm^2^) was 7.86 times that of UK (44.33 ± 7.23 mm^2^) in plasminogen-rich fibrin plates, and the fibrinolytic activity of FELP in plasminogen-rich fibrin plates was 2.63 times that of FELP (132.33 ± 11.5 mm^2^) in plasminogen-free fibrin plates.

### 2.6. Degradation of Fibrinogen In Vitro

**Figure 8:**
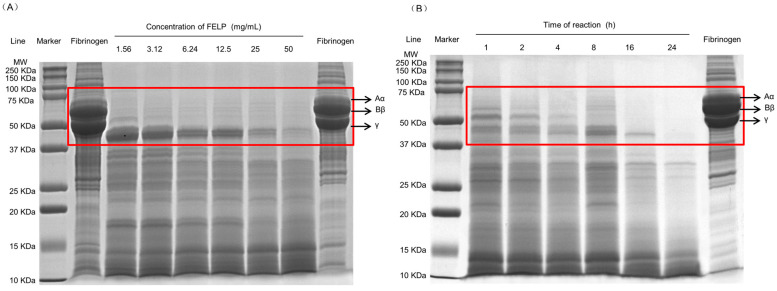
Fibrinogenolytic activity of FELP. (**A**) Degradation pattern of fibrinogen by FELP in a dose-dependent manner. Lanes: Fibrinogen on the left was unincubated. Fibrinogen on the right (10 mg/mL) incubated at 37 °C for 24 h. (**B**) Cleavage pattern of human fibrinogen by FELP (50 mg/mL) in a time-dependent manner. The Aα-, Bβ-, and γ- chains of fibrinogen were shown in the red rectangular frame.

To elucidate the degradation mechanism of fibrinogen by FELP, it was analyzed with 12.5% SDS-PAGE. FELP could hydrolyze the three chains of fibrinogen and showed dose and time dependency. As shown in Figure 8A, at low enzyme concentrations, the Aα-chain was completely degraded, and as the enzyme concentration increased, the Bβ-chain was gradually and completely degraded, while the γ-chain was the most difficult to hydrolyze and could not be completely degraded at the sample concentration of 50 mg/mL. As the reaction time increased (Figure 8B), the Aα-chain of fibrinogen was degraded first, followed by the Bβ-chain, which began to disappear, and after 24 h, the γ-chain was eventually hydrolyzed as well.

## 3. Discussion

Streptokinase, urokinase, pro-urokinase, reteplase, and alteplase are examples of fibrinolytic enzymes and thrombolytic drugs currently used in clinical settings. These drugs have significant unintended physiological side effects, including excessive bleeding, a short plasma half-life, limited fibrin specificity, and high therapeutic doses. As a vicarious therapy, traditional medicinal animals, such as earthworms, snakes, and leeches, have attracted more attention in the past few decades [26]. A novel fibrinolytic protein DPF3, which was purified from *Pheretima vulgaris*, possesses the potential to be developed into a promising antithrombotic agent [27]. The antithrombotic protein named EPf3, purified from *Pheretima guillelmi*, was found to confer excellent anticoagulant and thrombolytic activity and could be developed into a promising antithrombotic agent [28]. An earthworm known in Chinese medicine as “Di Long” has long been used in the form of dried powder in the treatment of diseases, and a fibrinolytic enzyme called lumbrokinase has been purified from the earthworm [29].

In this study, a plasmin-like marine protease was isolated and purified from Annelida (phylum), *P. aibuhitensis*, named FELP. It was purified to homogeneity by using two column chromatography steps with a purification of 10.61-fold, which demonstrates that the protease is a microcomponent in *P. aibuhitensis*. Compared with the traditional purification of fibrinolytic enzymes [30,31], the target protease with fibrinolytic activity could be purified in only two steps, which greatly improved the purification efficiency and had a wide range of application prospects. Moreover, in the purification of FELP with activity monitoring, the fractions are collected from the first peak of G-50 to the base regression level, and only one tube has a protein band, indicating that fraction i is single. Our results show that the molecular mass of the purified enzyme is estimated to be 28.9 kDa with SDS-PAGE, which is within the molecular weight range of most fibrinolytic enzymes [25]. The MW of the purified enzyme is similar to the fibrinolytic protein from annelids, such as *Pheretima vulgaris* [27], and snake venom [32]. Few proteases with fibrinolytic activity have been isolated and purified from marine beach organisms, demonstrating that FELP is a novel fibrinolytic enzyme and can be a potential source for the development of therapeutic agents for the clinical treatment of thrombosis.

Based on the mechanism of action, fibrinolytic enzymes are classified as serine proteases, metalloproteinases, and serine metalloproteinases; the inhibitor of the enzyme is crucial for studying the types of enzymes. To further explore the physicochemical properties of FELP, corresponding inhibitors were used to examine their effect on FELP activity. As shown in Figure 3, the activity of FELP is correlated with SBTI and PMSF; however, the activity of FELP had not been affected by EACA and EDTA. PMSF, known as a serine protease inhibitor, can be used to identify fibrinolytic enzymes of the serine protease group [33] by sulphonate essential serine residues in the active site of the protease, resulting in a complete loss of enzyme activity [34]. As we all know, t-PA and uPA are the only serine proteases currently approved by the US Food and Drug Administration. Most of the fibrinolytic proteases from the sandworm are serine proteases [35,36,37], while SBTI is a specific inhibitor for trypsin. The above results clarify that FELP is a trypsin-like serine protease.

As shown in Figure 4 and Figure 5, FELP is most active at pH 9.0 and can maintain good stability in an alkaline environment, suggesting that FELP may be an alkaline protease. Moreover, the optimum pH for FELP is higher than the protease from terrestrial organisms [27,38]. It was observed that FELP is stable at 30–60 °C and can still maintain more than 60% activity after incubation for 2 h, exhibiting excellent thermal stability. Its optimum temperature is 50 °C, which is higher than that of the NJP isolated from Neanthes japonica (Iznka) [35]. Furthermore, FELP has excellent fibrinolytic activity at 37 °C and neutral pH, which indicates that the enzyme can retain its fibrinolytic activity in the human body and has application prospects.

Due to the information provided by Uniprot, B8Y626 has serine-type endopeptidase activity. Also, all six peptide fragments have a protein group of one, indicating that these peptides are unique for this protein. The sequence homology results also show that the purified enzyme may belong to serine protease (Figure 6). The above studies have been cross-checked to indicate that FELP is a trypsin-like serine protease. The peptides obtained are as follows:

IVGGQESRPNEFPWQVSMQSSFGSHYCGAIIINRNWIMTAAHCTAGDSASDLYLMVGEHDR; TAVVSGWGTLRSGGPCCPQILQYVQVPVISNNECNTIDYPGDITDGMICAGNRLSTDACQGDSGGPLVVK; VSTYMNWIGL

The purified enzyme can be observed as a distinctly transparent circle on fibrin plates. As illustrated in Figure 7A, both FELP and UK exhibited strong fibrinolytic activity in plasminogen-rich plates; additionally, we used plasminogen-free plates. However, UK could not cleave plasminogen to formative plasmin to achieve fibrinolytic activity; as expected, it did not obtain a transparent circle in Figure 7B. Nevertheless, FELP also showed hydrolyzing activity in the plate without plasminogen. Therefore, we believe that FELP can not only directly hydrolyze fibrin but also convert plasminogen to plasmin; this suggests that FELP shows a bi-functional manner in hydrolyzing fibrin, as documented in the reports on a fibrinolytic protein from *Pheretima vulgaris* [27], a fibrinolytic enzyme from the marine-derived fungus *Aspergillus versicolor* ZLH-1 [17], and an antithrombotic protease from *Sipunculus nudus* [21]. The present study shows that FELP exhibits excellent fibrinolytic activity.

In addition, FELP can exert its fibrinolytic activity by degrading fibrinogen, and it shows a dose and time dependency. The fibrinogenolysis starts with the Aα-chain, followed by the Bβ-chain and the γ-chain. This pattern is similar to that observed with fibrinolytic enzymes from most annelids, such as hirudo [39] and *Pheretima vulgaris* [27]. The γ-chain is the most difficult to degrade and was not completely degraded after 5 h of incubation at the sample concentration of 50 mg/mL. The mixture of fibrinogen and FELP was incubated at 37 °C for 24 h, and the γ-chain was finally completely degraded. The fibrinogen without the addition of FELP did not show degradation with incubation at 37 °C for 24 h (Figure 8), suggesting that the degradation of the three chains of fibrinogen was entirely due to the addition of FELP. From the above discussion, it can be concluded that FELP may show strong fibrinolytic activity by degrading fibrinogen.

## 4. Materials and Methods

### 4.1. Chemicals and Reagents

Ammonium sulfate (NH_4_)_2_SO_4_), Tris (hydroxymethyl) aminomethane, agarose, and ethylene diamine tetraacetic acid (EDTA) were purchased from Sinopharm Chemical Reagent Co., Ltd. (Shanghai, China). Urokinase was purchased from Shanghai Macklin Biochemical Technology Co., Ltd. (Shanghai, China). Fibrinogen was purchased from Beijing Solarbio Science & Technology Co., Ltd. (Beijing, China). Thrombin was purchased from Abmole (Houston, TX, USA). An Omni-EasyTM One-step PAGE Gel Fast Preparation Kit (12.5%, Catalog No. PG213) was purchased from Shanghai Epizyme Biomedical Technology Co., Ltd. (Shanghai, China). A 10× Tris/Glycine/SDS Buffer (Catalog 1610732), 4× Laemmli Sample Buffer (Catalog No. 1610747) and Precision Plus Protein Dual Color Standards with MW of 10–250 kDa (Catalog 1610374) were purchased from Bio-Rad Laboratories Inc. (Hercules, CA, USA). The bicinchoninic acid (BCA) protein quantitation kit (Catalog PA115-01) was purchased from Tiangen Biotech (Beijing, China) Co., Ltd. (Beijing, China). DEAE-Sepharose FF, Sephadex G-50, soybean trypsin inhibitor (SBTI), and phenyl methane sulfonyl fluoride (PMSF) were purchased from Shanghai yuanye Bio-Technology Co., Ltd. (Shanghai, China). Epsilon amino caproic acid (EACA) was purchased from Shanghai Aladdin Biochemical Technology Co., Ltd. (Shanghai, China). All reagents used were of analytical grade unless otherwise stated.

### 4.2. Preparation of Crude Extracts of FELP from P. aibuhitensis

*P. aibuhitensis* was provided by Yancheng Fengyueyuan Fishing Bait Co., Ltd. (Huangshagang, China). The isolation of FELP from *P. aibuhitensis* was based on the method of Zhihui Deng et al. (2010) with modifications [35]. All operations were carried out at 4 °C. For specific operating procedures, refer to Ge et al. (2022) [40]. The 500 mg dried sample was immersed in 100 mL PBS for 3 h and centrifuged at 9900× *g* for 20 min at 4 °C using a Himac CR 21G high-speed floor centrifuge (Hitachi, Tokyo, Japan). Ammonium sulfate was stirred into the supernatant until dissolved, reaching 30 to 90% saturation, and then left for 2 h. The precipitates collected with centrifugation at 15,400× *g* for 30 min were resuspended in PBS (pH 7.4) and dialyzed using dialysis membranes (MWCO: 10 kDa) against 0.02 M Tris-HCl (pH 7.4) for 48 to 72 h. The dialyzed sample was pre-cooled at −80 °C for 4 h and lyophilized using a freeze-dryer (Labconco Freezone 2.5 L, Kansas City, MO, USA). The fibrinolytic activity and protein content of the samples taken at each ammonium sulphate saturation level were measured to calculate the specific activity.

### 4.3. The Fibrin-Plate Method

The fibrin-plate assay was used to study the fibrinolytic activity of FELP, according to Astrup and Müllertz [41] with slight modifications. Both fibrinogen-free and fibrinogen-rich plates were used. To prepare the plasminogen-rich fibrin plates, specifically speaking, 10 mL of a 0.2% (*w*/*v*) fibrinogen solution in saline (medicine) was mixed with 10 mL of a 1.5% (*w*/*v*) agarose solution and 1 mL of a thrombin solution (20 U/mL) in a Petri dish and then kept at ambient temperature for 1 h to form a fibrin clot layer. A total of 10 μL of sample solutions were carefully placed onto the pre-punched holes of the fibrin plate and incubated at 37 °C for 18 h. The fibrinogen-free fibrin plates were prepared by placing the fibrin plates at 80 °C for an additional 2 h to extinguish the fibrinogen. To determine fibrinolytic activity, FELP (50 mg/mL) was prepared; urokinase (1000 U) was used as a positive control, and saline (0.9% NaCl) was used as a negative control. The activity was quantified by measuring the area of fibrinolysis on the plate.

### 4.4. Protein Concentration

Protein concentration was measured with the BCA Protein Assay Kit. The principle is that the peptide bond structure in proteins can generate complexes with Cu^2+^ and reduce Cu^2+^ to Cu^+^ under an alkaline environment, and the BCA reagent can specifically bind to Cu^+^ to form a colored complex with maximum absorbance at 562 nm. The 96-well plate was spiked with 20 µL of sample; then, 200 µL of BCA working solution was added and incubated for 30 min at 37 °C. The absorbance at 562 nm was measured, wherein bovine serum albumin (BSA) was used as the standard.

### 4.5. Enzyme Purification

The crude enzyme (200 mg) was applied onto a DEAE Sepharose FF column (diameter: 2.6 cm, height: 20 cm) and equilibrated with 20 mM Tris-HCl buffer (pH 7.4). The bound proteins were eluted with 0.1 to 0.5 M NaCl in the 20 mM Tris-HCl buffer (pH 7.4) at a flow rate of 1 mL/min and were monitored with HD-3 (Shanghai Huxi Analysis Instrument Factory Co., Ltd., Shanghai, China) with an ultraviolet detector at 280 nm. The activity of the individual fractions was detected using the fibrin-plate assay. The active fractions (1 g) were pooled and further purified with Sephadex G-50 (diameter: 1.6 cm, height: 60 cm) and equilibrated with 20 mM Tris-HCl buffer (pH 7.4) at a flow rate of 0.8 mL/min with one tube collected every 3 min. The fraction with fibrinolytic activity was collected and lyophilized as the purified enzyme preparation.

### 4.6. SDS-PAGE

The molecular pattern of the FELP from *P. aibuhitensis* was determined by using SDS-PAGE (separating gel of 12.5% polyacrylamide with a stacking gel of 4.5% polyacrylamide) according to Laemmli’s method [42]. The mixture of the sample (50 mg/mL) and the 4× Laemmli Sample Buffer was boiled for 5 min with a palm-sized drive lock incubator, standard type (Bio Medical Science Inc., Tokyo, Japan) and then briefly centrifuged at 1800× *g* using an S1010E mini-centrifuge (SCILOGEX, Rocky Hill, CT, USA). The samples were placed at room temperature, and the Precision Plus Protein Dual Color Standards were loaded onto the lanes of the gel and then electrophoresed at a constant voltage of 180 V with 1× Tris/Glycine/SDS Buffer for 45 min using a Mini-PROTEAN Tetra Cell (Bio-Rad Laboratories Inc., Richmond, CA, USA). After the gel was discolored in a solution prepared with 0.25% (*w*/*v*) Coomassie Brilliant Blue R250 for 20 min, it was transferred to the decolorizing fluid (Ethanol:Acetic acid:H_2_O = 2:1:7) on a shaker with 80 rpm speed until clear protein bands were observed. Then, the gel was imaged with the GenoSens 2100 (T) Clinx Gel Documentation System (Clinx Science Instruments Co., Ltd., Shanghai, China).

### 4.7. Analysis of FELP from P. aibuhitensis

The sample was obtained from a clearly visible SDS-PAGE protein band and was analyzed with liquid chromatography–tandem mass spectrometry (LC–MS/MS). Specifically, the gel strip was cut after reduction and alkylation treatment and digested with trypsin at 37 °C for 20 h. The enzyme digestion products were desalted, lyophilized, and re-dissolved in 0.1% FA solution. The column was equilibrated with 95% liquid A (liquid A was an aqueous solution of 0.1% formic acid, and liquid B was an aqueous solution of 0.1% formic acid in acetonitrile (84%)). The mixture of peptides was loaded onto the trap column by the autosampler and separated with Easy-nLC 1000 (Thermo Fisher Scientific; Waltham, MA, USA). The peptides with high abundance were analyzed with a Q Exactive mass spectrometer (Thermo Fisher Scientific; Waltham, MA, USA); the primary mass resolution was 70,000 at 200 *m/z*, secondary mass spectral resolution was 17,500 at 200 *m/z*, normalized collision energy was 27 eV, and underfill was 0.1%. The data obtained from the secondary mass spectrometry were compared with the data retrieved from the corresponding database (Uniprotkb_Perinereis_245_2023_08_20) using the search engine Proteome Discoverer 2.5 to obtain the identified proteins. The three-dimensional structural homology modeling of FELP was projected with the SWISS-MODEL (https://swissmodel.expasy.org/, accessed on 22 January 2024) based on the amino acid sequence. The secondary structure of the peptides was predicted with the Protein Structure Prediction Server (PSIPRED) (http://bioinf.cs.ucl.ac.uk/psipred/, accessed on 22 January 2024).

### 4.8. Effect of Protease Inhibitors on Fibrinolytic Activity

FELP was incubated with PMSF (5 mM), EDTA (5 mM), SBTI (5 mM), and EACA (10 mM) at 37 °C for 2 h; a total of 10 μL of the mixture was removed to the already configured fibrin plate (the plate with a diameter of 4 cm) and incubated for 18 h to measure the two vertical diameters of the transparent circle to calculate the activities. The relative activity was calculated by dividing the enzyme activity under each condition by the enzyme activity under the condition without added inhibitors. The activity of FELP without inhibitors was considered to be 100%. Three parallel experiments were performed for each set of conditions.

### 4.9. Effect of Temperature on Fibrinolytic Activity and Thermal Stability

The influence of temperature on fibrinolytic activity was determined with the fibrin-plate assay. FELP was placed on the prepared fibrin plate, incubated at 30 to 65 °C for 18 h, and the vertical diameter of the transparent circle was measured to calculate the activities. The relative activity was calculated by dividing the activity for each condition by the maximum activity. FELP was incubated at temperatures of 30, 40, 50, 60, 70 and 80 °C; the 10 μL samples were collected at 0 min, 20 min, 40 min, 60 min, 80 min, 100 min, and 120 min to assess the thermal stability of the protease. The enzyme activity before holding was defined as 100%; the residual enzyme activity under the other conditions was calculated. Three parallel experiments were performed at each gradient.

### 4.10. Effect of pH on Fibrinolytic Activity and pH Stability of FELP

The effect of pH on fibrinolytic activity was recorded after incubation of FELP at a pH of 3, 4, 5, 6, 7, 8, 9, 10, and 11. The buffers were 0.1 M citric acid –sodium citrate buffer (pH 3–5), 0.1 M phosphate-buffered saline (pH 6–7), 0.1 M Tris-HCl (pH 8–9), and 0.1 M sodium carbonate–bicarbonate buffer (pH 10–11). FELP was redissolved in buffers of different pH; a total of 10 µL of each gradient was added to fibrin plates prepared with a different pH and incubated for 18 h at 37 °C. The relative activity was calculated by dividing the activity measured in each condition by the maximum activity. The pH stability of FELP was evaluated by incubating the enzyme in different buffers at intervals of 0 h, 2 h, 4 h, 6 h, 8 h, and 10 h. The enzyme activity before incubation was defined as 100%, and the residual enzyme activity under the other conditions was calculated. Three parallel experiments were performed at each gradient.

### 4.11. Degradation of Fibrinogen In Vitro

#### 4.11.1. Dose-Effect Evaluation

Fibrinogen degradation with FELP in a dose-manner was analyzed with SDS-PAGE. Briefly, 1% fibrinogen was mixed with different sample concentrations (1.56 mg/mL, 3.12 mg/mL, 6.24 mg/mL, 12.5 mg/mL, 25 mg/mL, and 50 mg/mL) of fibrinolytic enzymes and incubated at 37 °C for 5 h. The concentration of proteins was determined with the BCA Protein kit. Equal amounts of total proteins were resuspended in loading buffer and separated with 12.5% SDS-PAGE. Each gradient was repeated three times in independent experiments.

#### 4.11.2. Time-Effect Evaluation

The degradation pattern of fibrinogen by FELP was also analyzed with SDS-PAGE. Briefly, 100 μL of 1% fibrinogen was incubated with 100 μL (50 mg/mL) of FELP for 1 h, 2 h, 4 h, 8 h, 16 h, and 24 h, respectively. Each dosage was repeated in triplicate, and independent experiments were performed. After treatment, 30 µL of the samples was quantified with a BCA assay kit. Equivalent amounts of proteins were removed and mixed with 4× Laemmli sample buffer to stop the reactions. The samples with the buffer were boiled at 100 °C for 5 min, brought to room temperature, and stored at 4 °C to standby. The products were separated with 12.5% SDS-PAGE.

### 4.12. Statistic Analysis

Statistical analysis was performed using GraphPad Prism 9 software (GraphPad Inc, San Diego, CA, USA) using two-way ANOVA with Fisher’s LSD test multiple comparison analysis. Data were presented as means ± SD. The values identified as outliers were excluded from the statistical analysis. The results were considered statistically significant if the *p*-value < 0.05.

## 5. Conclusions

In this study, FELP from *P. aibuhitensis* was purified, and its physicochemical properties and activity in vitro were studied. The molecular weight of FELP was found to be 28.9 kDa. Fibrin-plate assays showed the enzyme was a fibrinolytic protease, which can exert fibrinolytic activity by directly acting on fibrin or through activation of fibrinogen. Inhibition of fibrinolytic activity by the standard serine protease inhibitors PMSF and SBTI confirmed it to be a trypsin-like serine protease. The peptide fragments were characterized with LC–MS/MS. The enzyme showed maximum fibrinolytic activity at pH 9 and temperature 50 °C and has excellent stability at pH 7–11 or 30–60 °C. FELP exhibited higher fibrinolytic activity than urokinase (1000 U) in fibrin plates and not only directly dissolved fibrin but also indirectly exerted fibrinolytic activity through the activation of fibrinogen. The gel electrophoresis analysis results of the fibrinogenolytic mechanism showed that the degradation of the subunit in fibrinogen demonstrated dose and time dependency, which was in the order chain A-α, chain B-β, and chain γ, and three chains were nearly thoroughly degraded after 24 h. Collectively, our results identified a marine-derived enzyme. We were surprised to find that it has shown stable fibrinolytic activity, but its thrombolytic mechanism needs to be further studied and verified.

## Figures and Tables

**Table 1:** Purification table of FELP from *P. aibuhitensis*.

Purification Steps	Purification Media	Activity (U) ^1^	Protein (μg) ^2^	Specific Activity (U/μg)	Purification Folds
Crude isolation	Ammonium sulphate precipitation	7894.06	170.21	46.38	1.00
Ion-exchange chromatography	DEAE- Sepharose FF	677.42	4.52	150.00	3.23
Gel-filtration chromatography	Sephadex G-50	1398.14	2.84	492.30	10.61

^1^ Activity was measured in 1 mg of the sample at each purification step; ^2^ Protein was measured in 1 mg of the sample at each purification step.

**Table 2:** Peptide fragments were obtained by comparison with the database.

Sequence	Proteins	Missed Cleavages	Ions Score
[R].NWIMTAAHCTAGDSASDLYLMVGEHDR.[S]	1	0	58
[R].IVGGQESRPNEFPWQVSMQSSFGSHYCGAIIINR.[N]	1	0	51
[R].LSTDACQGDSGGPLVVK.[D]	1	0	94
[R].VSTYMNWIGL.[-]	1	0	9
[R].SGGPCCPQILQYVQVPVISNNECNTIDYPGDITDGMICAGNR.[L]	1	0	49
[R].TAVVSGWGTLR.[S]	1	0	58

## Data Availability

The original data presented in the study are included in the article; further inquiries can be directed to the corresponding author.
